# The American Medical Association

**Published:** 1867-06

**Authors:** 


### THE AMERICAN MEDICAL ASSOCIATION.

THIS, the most important medical society in America, lately met in this city. We were prevented from giving much attention to its meetings. Some of its proceedings were highly important, some not very, as appears to be necessary in this fallen condition of mankind. The profession here, and the city, feasted the members, showed them the fire engines, took them riding on a steamboat and in carriages, took them to a wine-cellar, to the lunatic asylum, and to a grave-yard. The whole was a grand scene of huge enjoyment; and what is remarkable, the profession here had a little money left in their treasury, after defraying the expenses of all the entertainments.

Prof. Gross, of Philadelphia, was president. The next meeting will be in Washington City. A few subjects specially interesting to Dentists were discussed, but we are not sufficiently posted to speak, in detail, of their disposition. We presume the members feel about as those of the "American Dental Association" usually do on such occasions, that so much of display, entertainment, and hospitality in general, while very pleasant, interferes very much with the scientific results of the meeting. But this is a difficult matter to regulate. Some people like to be hospitable, or at least dislike to be far behind their neighbors in this respect. We believe the wish of the Association is to have less time, hereafter, devoted to the social, and more to the scientific interests of the profession; and, as we are imitative animals, we hope, after the Cincinnati meeting, the Dental Association will follow suit. We can eat and drink, and "laugh and grow fat" at home; but we cannot there interchange scientific thoughts with the leading minds of the profession, within the space of a few days. W.

